# Soil-transmitted helminth infections, anemia and undernutrition among schoolchildren in Yirgacheffee, South Ethiopia

**DOI:** 10.1186/s13104-018-3679-9

**Published:** 2018-08-13

**Authors:** Eshetu Molla, Hassen Mamo

**Affiliations:** 1 Department of Medical Laboratory Sciences, College of Health Sciences and Medicine, Dilla University, PO Box 419, Dilla, Ethiopia; 2 Department of Microbial, Cellular and Molecular Biology, College of Natural Sciences, Addis Ababa University, PO Box 1176, Addis Ababa, Ethiopia

**Keywords:** Soil-transmitted helminth, Anaemia, Nutritional status, Association, Schoolchildren, Kato–Katz

## Abstract

**Objective:**

Current data on soil-transmitted helminth infections, anemia and malnutrition that are largely neglected is vital to the control and management of them in a specific setting. This study was, therefore, aimed at determining the status of the three health concerns in one of the high-risk groups, schoolchildren, in South Ethiopia.

**Results:**

Among the 443 sampled schoolchildren, 54% were infected with soil-transmitted helminths (STHs) and 15.4% of them had anaemia, while the prevalence rate of undernutrition was 28.9%. Species-wise, prevalence of STH infections was 21.7, 16.7, 7.2 and 8.4% for *Ascaris lumbricoides,* the hookworms, *Trichuris trichiura* and mixed infections, respectively. Untreated drinking water, high frequency of sucking fingernails and open defecation were significantly associated with risk of getting STH infections. Child positivity for STH infection didn’t show any significant association with undernutrition of the children. Anaemia was significantly correlated with hookworm (adjusted odds ratio (AOR) = 2.96, 95% confidence interval (CI) = 2.15, 4.86), *A. lumbricoides* (AOR = 1.93, 95% CI = 1.13, 3.01) and polyparasitism (AOR = 1.54, 95% CI = 1.04, 2.64). In addition, children with heavy intensities of hookworm infections and those undernourished were more likely to suffer from anaemia with P = 0.001 and P = 0.007, respectively.

## Introduction

Soil-transmitted helminth (STH) infections are common parasitic infections in the tropics and subtropics [1, 2]. More than 568 million school-age children live in areas where these parasites are intensively transmitted, and are in need of treatments. Although the global target is to eliminate morbidity due to STH infections in children by 2020 [2], these infections are still huge health problems in Ethiopia affecting millions of school-children. The prevalence may reach up to 83% among residents in some rural regions of the country [3].

Anaemia, is another global public health issue, affects around 40% school-age children in developing world [4, 5]. Previous reports revealed that anaemia in school-aged children is strongly associated with moderate and heavy hookworm infections [6–8]. But, other studies indicated the presence of STH infections did not significantly associate with low haemoglobin (Hb) levels [9–11], warranting further study in different settings.

Child undernutrition (mainly involving stunting, wasting and underweight) has also been a serious global public health problem in developing world including Ethiopia [12]. In Ethiopia, the magnitude of childhood undernutrition has decreased from 58% in 2000 to 40% in 2014, but it is sustained as the major public health concern causing repetitions in primary schools [13].

Although STH infections, anemia and undernutrition are the major public health problems in Ethiopia, in general [14] and in the current study area [15], in particular; data on them among schoolchildren are scarce. Such lack of information hinders the planning of appropriate interventions [14]. Additionally, in the country, there are conflicting reports regarding the association of these helminth infections with anaemia and undernutrition. Hence, it is necessary to investigate the status and their relationship to each other of these health concerns to generate data that would guide to evaluate the existing programs and establish new intervention measures. Thus, the aim of this study was to evaluate the current prevalence of STHs, anaemia and nutritional status and their relationships to each other among schoolchildren in selected elementary schools.

## Main text

### Methods

#### Study design and area

School based cross-sectional study design was employed in Yirgacheffee district elementary schools, South Ethiopia from October to November, 2017. There are 36 schools in both in rural and urban areas of the district. It is estimated that over 7100 schoolchildren were following their education in these schools [16].

#### Study population

Children aged 6–15 years old, not terminally ill, having no any other disease conditions, not on anti-helminthic chemotherapy for the past 4 weeks and with no iron supplementation were included in the study.

#### Sampling

A total of 448 schoolchildren were recruited to participate in the study. The sample size was calculated using single population proportion formula (P = 0.107 [17], Z_α/2_ = 0.196, d = 0.03, confidence interval (CI) = 95% and 10% non-response rate). The study was comprised of 6 randomly selected schools from the total of 36 schools of the district. A simple random sampling technique was used to select the schools from both areas (rural and urban). Accordingly, 3 schools were randomly selected from each area. Systematic random sampling technique was employed to select the sampling unit by using class roster (in the school registry).

#### Parasitological examination

Fresh stool samples were collected in small clean labeled plastic containers. Then, Kato–Katz slides were prepared within 2 h as per WHO protocol. Briefly, a portion of the fecal specimen was taken by clean wooden spatula and forced through a nylon screen. The screened fecal material was transferred to the template which was laid flat centrally on a microscope slide. The template hole was completely filled with screened fecal material and leveled to the surface of the template. Malachite green-glycerin soaked cellophane square and cellophane tape were employed for further procedures. The prepared Kato–Katz slides were examined under microscope for STHs ova. Eggs were counted for all STH species to record eggs per gram of faeces (EPG). EPG of faeces for each species of parasites were obtained when average of two slides egg counts multiplied by a conversion factor of 24 [18]. Intensity of infection was interpreted as light, moderate or heavy, as described by WHO’s Expert Committee criteria [19]. Duplicate Kato–Katz slides were prepared from each stool specimen and all were examined twice by two different laboratory technologists. Moreover, negative samples were re-examined on the same day at the same time by another laboratory technician.

#### Hb level assessment

Blood samples were collected by finger pricks (approx. 10 μl) using disposable lancet and Hb levels were measured by using the Hemocue haemoglobinometer (Hemocue HB 301 analyzer, Ängelholm, Sweden). The tip of ring finger was cleaned with alcohol-soaked cotton and pricked with a blood lancet, and two drops of blood were wiped away with dry cotton. Then, the next drops of blood samples were used to fill the micro-cuvette. The filled micro-cuvette was then put on the holder and pushed into the HemoCue instrument. The Hb value was displayed in g/dl after approximately 30 s were then recorded. All children with Hb levels lower than 11.5 g/dl were considered as anaemic [20].

#### Nutritional status measurement

Anthropometric measurements were assessed in bare foot and school uniform, in children who were infected with the target helminths. Then, Z-values for weight-for-age (WAZ), body-mass-index-for-age (BAZ) and height-for-age (HAZ) were calculated. These Z-tests of proportions were then used to test the prevalence of stunting, underweight and wasting. A child with Z-values of BAZ, HAZ and WAZ < − 2 was grouped as undernutrition [21]. Finally, socio-demographic and predisposing factors information were collected using semi-structured questionnaire.

#### Data analysis

The collected data were checked for their completeness manually and then entered into Epi Info version 3.1 and then exported to SPSS version 20.0 (SPSS, IBM, Chicago, USA) for analysis. Descriptive statistics and univariate analysis were performed to describe the population demographics and to assess the prevalence of the three health problems. The intensity of helminth infection was expressed as geometric mean. A bivariate logistic regression model analysis was done to see the association between the explanatory and outcome variables including among age groups, gender, and other risk factors of STH infections and anaemia. Thereafter, multivariable logistic regression analysis was performed by selecting candidate variables to STH infections and anaemia with P-value ≤ 0.2 in the bivariate analysis. Odds ratio with 95% CI was used to measure the strength between dependent and independent variables at P-value < 0.05 to declare the level of statistical significance.

### Results

A total of 443 (with 98.7% response rate) schoolchildren were recruited in the present study. The majority (53.0%) of study participants were from rural areas. Age of the children ranged from 6 to 15 years and the majority of them were in the age groups of 6–8 (34.3%). Over half (55.3%) of them were males and the predominant (59.8%) of the households had family size between 4 and 6 years. About 55.1% of them accessed treated water (Tables 1, 2).

**Table 1:** Prevalence STH infection, anaemia and under nutrition among schoolchildren in Yirgacheffee selected elementary schools, South Ethiopia, 2017

Variable	Rural, no. infected (%)	Urban, no. infected (%)	Total no. infected/positive (%)	P-value
STH infection				
*A. lumbricoides*	51 (53.1)	45 (46.9)	96 (21.7)	0.004
Hookworm	40 (54.1)	34 (45.9)	74 (16.7)	
*T. trichiura*	15 (46.9)	17 (53.1)	32 (7.2)	
Mixed infection	21 (56.8)	16 (43.2)	37 (8.4)	
	127 (53.1)	112 (46.9)	239 (54.0)	
Anaemia				
Yes	38 (55.9)	30 (44.1)	68 (15.4)	0.461
No	197 (52.5)	178 (47.5)	375 (84.6)	
Undernutrition				
Yes	66 (51.6)	62 (48.4)	128 (28.9)	0.275
No	167 (53.0)	148 (47.0)	315 (71.1)	
Types of undernutrition				
Stunting	25 (52.1)	23 (47.9)	48 (10.8)	0.089
Wasting	9 (39.1)	14 (60.9)	23 (5.2)	
Underweight	32 (56.1)	25 (43.9)	57 (12.9)	

*STH* soil-transmitted helminth, *no.* number

**Table 2:** Association of STHs and anaemia with selected risk factors among schoolchildren (N = 443) in Yirgacheffee selected schools, South Ethiopia, 2017

Socio-demographic variables	Total N (%)	STH		P-value	Anaemia		AOR (95% CI)	P-value
		Positive n (%)	Negative n (%)		Yes	No		
Gender								
Male	245 (55.3)	131 (54.8)	114 (55.9)		31	213	1.0	
Female	198 (44.7)	108 (45.2)	90 (44.1)	0.286	37	161	1.90 (0.72, 3.02)	0.47
Age groups								
6–8	152 (34.3)	94 (21.2)	58 (13.1)		21	125	1.0	
9–11	142 (32.1)	70 (15.8)	72 (16.3)	0.054	27	136	1.39 (0.94, 2.72)	
12–15	149 (33.6)	75 (16.9)	74 (16.7)		20	114	1.21 (0.67, 1.93)	0.63
Source of water								
Untreated	199 (44.9)	127 (63.8)	72 (36.2)	0.000	NA	NA		
Treated	244 (55.1)	112 (45.9)	132 (54.1)		NA	NA	–	–
Frequency of sucking fingers/nails								
Always	120 (27.1)	79 (65.8)	41 (34.2)		NA	NA	–	–
Sometimes	226 (51.0)	96 (42.5)	130 (57.5)	0.000	NA	NA		
Never	54 (12.2)	41 (75.9)	13 (24.1)		NA	NA		
Don’t know	43 (9.7)	23 (53.5)	20 (46.5)		NA	NA		
Place of defecation								
Open field	148 (33.4)	89 (60.1)	59 (39.9)	0.040	NA	NA	–	–
Toilet	295 (66.6)	150 (50.8)	145 (49.2)		NA	NA		
Family size								
1–3	79 (17.8)	30 (38.0)	49 (62.0)		NA	NA	–	–
4–6	265 (59.8)	132 (49.8)	133 (50.2)	0.221	NA	NA		
> 7	99 (22.4)	77 (77.8)	22 (22.2)		NA	NA		
Undernutrition								
No	315 (71.1)	172 (54.6)	143 (45.4)		214	101	1.0	
Yes	128 (28.9)	67 (52.3)	61 (47.7)	0.213	83	45	2.95 (1.68, 4.81)	0.007

*AOR* adjusted odds ratio, *CI* confidence interval*, NA* not applicable

#### Prevalence findings

From the total sampled population, the overall prevalence of STH infection, anemia and undernutrition were 54.0%, 15.4% and 28.9%, respectively. The predominant helminths species was *A. lumbricoides* (21.7%) and the most prevalent form of undernutrition was underweight (12.9%) (Table 1).

#### Associations of selected risk factors with STH and anaemia

STH infections showed significant associations with source of drinking water, place of defecation and frequency of sucking fingers/nails (P < 0.05). There were no significant associations of STH infections with age, gender, family size and undernutrition (P > 0.05). Anaemia was significantly higher among children who were undernourished (AOR = 2.95, 95% CI 1.68, 4.81). STH infected children were more likely to be undernourished but this association was not significant (P = 0.213). In general, there were no significant associations of STH infections with age, gender and malnutrition. As well, the occurrence of anaemia was not statistically associated with age and gender (P > 0.05) (Table 2).

#### The association between STH and anaemia

As shown in Table 3, participants who infected with STH infection were at higher risk for anaemia than non-infected (AOR = 3.72, 95% CI 2.68, 5.34). *A. lumbricoides*, hookworm and mixed infections were associated with an increased risk of anaemia with (AOR = 1.93, 95% CI 1.13, 3.01), (AOR = 2.96, 95% CI 2.15, 4.86) and (AOR = 1.54, 95% CI 1.04, 2.64), respectively. Anaemia was significantly higher among children on those with an increase in hookworm intensity (AOR = 4.04, 95% CI 2.20, 6.17). But, the intensities of other STH infections didn’t show any significant association with anaemia.

**Table 3:** The association between individual STH infection and anaemia among schoolchildren in Yirgacheffee selected elementary schools, South Ethiopia, 2017

Variable	Have anaemia		AOR (95% CI)	P-value
	Yes (%) (Hb < 11.5 g/dl)	No (%) (Hb > 11 g/dl)		
STH infection				
Yes (239)	56 (23.4)	183 (76.6)	3.72 (2.68, 5.34)	0.006
No (204)	12 (5.9)	192 (94.1)		
Parasite species				
*A. lumbricoides*	17 (3.8)	75 (16.9)	1.93 (1.13, 3.01)	0.004
*T. trichiura*	3 (0.70)	27 (6.1)	1.42 (0.81, 2.17)	0.163
Hookworm	21 (4.7)	50 (11.3)	2.96 (2.15, 4.86)	0.002
Mixed infection	15 (3.4)	19 (4.3)	1.54 (1.04, 2.64)	0.019
Egg intensity in EPG				
*A. lumbricoides*	711.8	NA	0.31 (0.20, 1.02)	0.97
*T. trichiura*	504.7	NA	0.92 (0.51, 1.07)	0.26
Hookworm	810.4	NA	4.04 (2.20, 6.17)	0.001

*AOR* adjusted odds ratio, *CI* confidence interval, *EPG* eggs per gram of faeces, *Hb* Haemoglobin, *NA* not applicable

### Discussion

This prevalence of STH infection (54.0%) signified that STH remains still a public health concern among the study subjects in the study area. This estimate is comparable with results of similar studies elsewhere in Ethiopia [22–24]. On the other hand, the present prevalence rate is higher than the reports from different areas [6, 11, 25, 26]. By contrast, the prevalence of STH infection in the current study was lower than the reports in different parts of Ethiopia including in Zegie Peninsula (69.1%) [27], northwestern Ethiopia (66.4%) [28] and Zarima town (82.4%) [29]. Differences in socio-demographic/economic features and awareness levels towards STH infection exposures, transmissions and prevention might be determinant factors for the discrepancies of STH prevalence in the present study area as compared to the above reports.

With regard to species-wise prevalence, *A. lumbricoides* was predominant (21.7%) followed by hookworm (16.7%) and *T. trichiura* (7.2%). This prevalence found out in the current study is similar with the study conducted in Lake Ziway for *A. lumbricoides* [30].

The prevalence of anaemia was 15.4% in this study, which does not markedly differ from other studies carried out in northern Ethiopia (11%) [31], north-western Uganda 11.8% [32] and northwestern Morocco (16.2%) [33]. But it was lower than the reports from Shimbit, Ethiopia (25.8%) [6]; Jimma, Ethiopia (37.6%) [4]; Kenya (28.8%) [34]; Rwanda (30.9%) [35] and Upper Egypt (59.3%) [36]. But this rate was higher when compared to one study in northwest Ethiopia [37]. In addition, the 2016 Demographic and Health Survey of Ethiopia reported that around 56% of children and 23% of women suffered from anaemia [13], implying the current estimate of anemia in children was varying to what is reported in the above survey. These discrepancies can be justified by difference in sample size, the food consumption, variations in geographical locations, household wealth differences and behavioral differences across these different settings.

In this research, it was observed that source of drinking water, place of defecation and frequency of sucking fingers/nails showed significant associations with the development of STH infections. Other investigations have also been showed consistent result [7, 25, 26, 36].

From the total of investigated schoolchildren, 28.9% were suffered from undernutrition, from which the majority were affected by underweight. This finding is congruent to what was recorded in northwestern Ethiopia [17]; southern Ethiopia [38] and Khartoum, Sudan in the case of stunting [39]. In contrast, the prevalence of the three types of undernutrition in this study area was lower than the estimates found in other parts of Ethiopia and abroad [40, 41]. This discrepancy may be due to differences in the dietary diversity of children, socio-economic status, the difference in STH infection frequencies and intensities and other factors predisposing to undernutrition. In this study undernutrition was not statistically associated with STH infections. Congruent finding was documented by other researcher in northwest Ethiopia [17].

The associations of STH infections with anaemia in this finding showed children infected with A. lumbricoides, hookworm and mixed infections were more likely to develop anaemia. These infections can directly or indirectly affect Hb level, which might have led to iron deficiency anaemia. For example, the hookworms ingest blood, and damage the intestinal wall causing bleeding [42], while *A. lumbricoides* affects Hb level indirectly by reducing appetite and nutrition uptake in the intestine and obstructing the jejunum [7]. While some previous studies observed significantly higher prevalence of anaemia only among hookworm-positive cases [17, 43], others found both hookworm and ascaris to be associated with anemia [7, 8]. However, other studies have documented that presence of hookworm and ascaris infections [9], ascaris and trichuris infections [17] as well as all of the three STHs [11] did not significantly associate with reduction in Hb values.

This study confirmed that undernutrition and high intensity of hookworm infection were associated with an increased risk of anaemia among children. However, age and gender differences as well as the other two STH intensities did not increase the risk of anaemia. In agreement with the present report, some studies in Ethiopia revealed consistent reports [8, 43], but this goes not in line with findings from Kenya [34] and Morocco [33], in which they found that age and sex had association with anaemia.

### Conclusions

This finding revealed high prevalence of STH and undernutrition and relatively moderate level of anaemia among schoolchildren. Risk of STH infection was increased with untreated drinking water, open defecation and with frequent sucking fingernails. Anaemia was significantly associated with *A. lumbricoides*, hookworm, and mixed infections. Moreover, anaemia was associated with undernutrition and intensity of hookworm infection. The findings warrant the need to address the problem of undernutrition and also deworming the children.

## Limitations of the study

One of the limitations of this study stems from the study design, cross-sectional, where it may not indicate the cause and effect relationship of undernutrition, anaemia and STH infection. Moreover, the negative impact of STHs infection on children’s educational performance was not assessed. Hence, this finding should be interpreted by taking into account these limitations.

## Abbreviations

AOR: adjusted odds ratio
BAZ: body-mass-index-for-age
CI: confidence interval
DHS: Demographic and Health Survey
EPG: eggs per gram of faeces
HAZ: height-for-age
Hb: haemoglobin
STH: soil-transmitted helminths
WAZ: weight-for-age

## References

[1] WHO. Intestinal worms. http://www.who.int/intestinal_worms/epidemiology/en/. Accessed 2018.

[2] WHO. Soil-transmitted helminth infections, fact sheet. http://www.who.int/mediacentre/factsheets/fs366/en/. Accessed 2017.

[3] Negussu N, Mengistu B, Kebede B, Deribe K, Ejigu E, Tadesse G (2017). Ethiopia schistosomiasis and soil-transmitted helminths control programme: progress and prospects. Ethiop Med J.

[4] Assefa S, Mossie A, Hamza L (2014). Prevalence and severity of anemia among school children in Jimma Town, southwest Ethiopia. BMC Hematol..

[5] WHO. Anaemia. http://www.who.int/topics/anaemia/en/. Accessed 2018.

[6] Getnet A, Worku S (2015). The association between major helminth infections (soil-transmitted helminthes and schistosomiasis) and anaemia among school children in Shimbit elementary school, Bahir Dar, northwest Ethiopia. Am J Health Res..

[7] de Gier B, Nga TT, Winichagoon P, Dijkhuizen MA, Khan NC, van de Bor M (2016). Species–specific associations between soil-transmitted helminths and micronutrients in Vietnamese school children. Am J Trop Med Hyg.

[8] Gutema B, Adissu W, Asress Y, Gedefaw L (2014). Anemia and associated factors among school-age children in Filtu town, Somali region, southeast Ethiopia. BMC Hematol..

[9] Darlan DM, Ananda FR, Sari MI, Arrasyid NK, Sari DI (2018). Correlation between iron deficiency anaemia and intestinal parasitic infection in school-age children in Medan. IOP EES..

[10] Arrasyid NK, Sinambela MN, Tala ZZ, Darlan DM, Warli SM (2017). Correlation between soil-transmitted helminths infection and serum iron level among primary school children in Medan. Open Access Maced J Med Sci..

[11] Adewale B, Adedeji A, Folorunsho S, Demehin P, Akinsanya B (2017). Soil-transmitted helminth infections and risk factors among primary school pupils in Lagos, Nigeria. BMJ Glob Health.

[12] Tariku A, Woldie H, Fekadu A, Adane A, Ferede A, Yitayew S (2016). Nearly half of preschool children are stunted in Dembia district, northwest Ethiopia: a community based cross sectional study. Arch Public Health..

[13] Central Statistical Agency (CSA) [Ethiopia] and ICF. Ethiopia Demographic and Health Survey 2016: Key Indicators Report. Addis Ababa, Ethiopia, and Rockville, Maryland, USA. CSA and ICF; 2016. https://dhsprogram.com/pubs/pdf/FR328/FR328.pdf. Accessed July 2017.

[14] Mahmud MA, Spigt M, Bezabih AM, Lo pez Pavon I, Dinant G, Velasco RB (2013). Risk factors for intestinal parasitosis, anaemia and malnutrition among school childen in Ethiopia. Pathog Glob Health..

[15] Yirgacheffee District Health Report. Yirgacheffee district annual health report. Unpublished report. 2017.

[16] Gedeo Zone Education Office. Gedeo zone education office report. 2017.

[17] Alelign A, Degarege A, Erko B (2015). Prevalence and factors associated with undernutrition and anaemia among school children in Durbete, northwest Ethiopia. Arch Public Health..

[18] WHO. Basic laboratory methods in medical parasitology. Geneva: WHO; 1991. http://www.who.int/malaria/publications/atoz/9241544104_part1/en/. Accessed 1991.

[19] WHO. Prevention and control of schistosomiasis and soil-transmitted helminthiasis. Report of WHO expert committee. WHO Tec Rep Se No. 912: 63. http://www.who.int/intestinal_worms/resources/who_trs_912/en/. Accessed 2002. 12592987

[20] WHO. Iron deficiency anaemia: assessment, prevention and control. A guide for programme managers. Geneva: World Health Organization; 2001. http://www.who.int/nutrition/publications/micronutrients/anaemia_iron_deficiency/WHO_NHD_01.3/en/. Accessed 2001.

[21] WHO. Child Growth Standards: length/height-for-age, weight-for-age, weight-for-length, weight-for-height and body mass index-for-age. In: Methods and development. Geneva: WHO: 2006.

[22] Alelign A, Degarege A, Erko B (2015). Soil-transmitted helminth infections and associated risk factors among schoolchildren in Durbete town, northwestern Ethiopia. J Parasitol Res..

[23] Debalke S, Worku A, Jahur N, Mekonnen Z (2013). Soil transmitted helminths and associated factors among schoolchildren in government and private primary school in Jimma town, southwest Ethiopia. Ethiop J Health Sci..

[24] Degarege A, Animut A, Medhin G, Legesse M, Erko B (2014). The association between multiple intestinal helminth infections and blood group, anaemia and nutritional status in human populations from Dore Bafeno, southern Ethiopia. J Helminthol..

[25] Samuel F, Demsew A, Alem Y, Hailesilassie Y (2017). Soil transmitted helminthiasis and associated risk factors among elementary school children in Ambo town, western Ethiopia. BMC Public Health..

[26] Kumar DD, Kumari KR, James JK, Sekharan CB (2016). Soil-transmitted helminth infections and the associated risk factors in pre-primary school children, Kiwangwa rural ward, Bagamoyo District, Tanzania. Asian J Med Pharm Res..

[27] Abdi M, Nibretb E, Munshea A (2017). Prevalence of intestinal helminthic infections and malnutrition among schoolchildren of the Zegie Peninsula, northwestern Ethiopia. J Infect Public Health..

[28] Gashaw F, Aemero M, Legesse M, Petros B, Teklehaimanot T, Medhin G (2015). Prevalence of intestinal helminth infection among school children in Maksegnit and Enfranz towns, northwestern Ethiopia, with emphasis on *Schistosoma mansoni* infection. Parasit Vectors..

[29] Alemu A, Atnafu A, Addis Z, Shiferaw Y, Teklu T, Mathewos B (2011). Soil transmitted helminths and *Schistosoma mansoni* infections among school children in Zarima town, northwest Ethiopia. BMC Infect Dis.

[30] Teklemariam D, Legesse M, Degarege A, Liang S, Erko B (2018). *Schistosoma mansoni* and other intestinal parasitic infections in schoolchildren and vervet monkeys in Lake Ziway area, Ethiopia. BMC Res Notes..

[31] Mahmud MA, Spigt M, Bezabih AM, Pavon IL, Dinant G, Velasco RB (2013). Risk factors for intestinal parasitosis, anaemia, and malnutrition among school children in Ethiopia. Pathog Glob Health..

[32] Legason ID, Atiku A, Ssenyonga R, Olupot-Olupot P, Barugahare JB (2017). Prevalence of anaemia and associated risk factors among children in northwestern Uganda: a cross sectional study. BMC Hematol..

[33] Achouri I, Aboussaleh Y, Sbaibi R, Ahami A, El Hioui M (2015). Prevalence of iron deficiency anaemia among school children in Kenitra, Northwest of Morocco. Pak J Biol Sci.

[34] Ngesa O, Mwambi H (2014). Prevalence and risk factors of anaemia among children aged between 6 months and 14 years in Kenya. PLoS ONE.

[35] Angel MD, Berti P, Siekmans K, Tugirimana PL, Boy E (2017). Prevalence of iron deficiency and iron deficiency anemia in the northern and southern provinces of Rwanda. Food Nutr Bull..

[36] Salama RA, Labib MR (2016). The prevalence of anemia among informal primary school children: a community based study in rural upper Egypt. Epidemiol Biostat Public Health..

[37] Feleke BE, Derbie A, Zenebe Y, Mekonnen D, Hailu T, Tulu B (2017). Burden and determinant factors of anemia among elementary school children in northwest Ethiopia: a comparative cross sectional study. Afr J Infect Dis..

[38] Zerdo Z, Yohanes T, Tariku B, Teshome T (2017). Association between nutritional status and soil-transmitted helminthes re-infection among school-age children in Chencha district, southern Ethiopia: a cross-sectional study. Transl Biomed..

[39] Musa TH, Ali EA, Musa HH, Khan A (2013). Anthropometric parameters of malnutrition in children 5–15 years old in Khartoum State, Sudan. J Public Health Epidemiol..

[40] Degarege D, Degarege A, Animut A (2015). Under nutrition and associated risk factors among school age children in Addis Ababa, Ethiopia. BMC Public Health..

[41] Papier K, Williams G, Luceres-Catubig R, Ahmed F, Olveda R, McManus D (2014). Childhood malnutrition and parasitic helminth interactions. Clin Infect Dis.

[42] Sodhi JS, Khan MA (2016). Hookworm related obscure overt gastrointestinal bleeding diagnosed by capsule endoscopy. Int J Adv Res..

[43] Yimam Y, Degarege A, Erko B (2016). Effect of anthelminthic treatment on helminth infection and related anaemia among school-age children in northwestern Ethiopia. BMC Infect Dis.

